# Case volume and specialization in critically ill emergency patients: a nationwide cohort study in Japanese ICUs

**DOI:** 10.1186/s40560-024-00733-3

**Published:** 2024-05-17

**Authors:** Jun Fujinaga, Takanao Otake, Takehide Umeda, Toshio Fukuoka

**Affiliations:** 1 Emergency and Critical Care Center, Kurashiki Central Hospital, 1-1-1 Miwa, Kurashiki City, Okayama 710-8602 Japan; 2 Department of Epidemiology, Graduate School of Medicine, Dentistry and Pharmaceutical Sciences, Okayama University, 2-5-1 Shikata-Cho, Kita-Ku, Okayama, 700-8558 Japan

**Keywords:** Case volume, Critically ill patient, Emergency care, In-hospital mortality, Nationwide cohort study

## Abstract

**Background:**

Previous studies have explored the association between the number of cases and patient outcomes for critical illnesses such as sepsis and trauma, as well as various surgeries, with the expectation that a higher number of cases would have a more favorable effect on patient outcomes. The aim of this study was to elucidate the association among intensive care unit (ICU) case volume, specialization, and patient outcomes in critically ill emergency patients and to determine how ICU case volumes and specializations impact the outcomes of these patients in Japanese ICUs.

**Methods:**

Utilizing data from the Japanese Intensive Care PAtient Database (JIPAD) from April 2015 to March 2021, this retrospective cohort study was conducted in 80 ICUs across Japan and included 72,214 emergency patients aged ≥ 16 years. The primary outcome measure was in-hospital mortality, and the secondary outcomes encompassed ICU mortality, 28-day mortality, ventilator-free days, and the lengths of ICU and hospital stays. Bayesian hierarchical generalized linear mixed models were used to adjust for patient- and ICU-level variables.

**Results:**

This study revealed a significant association between a higher ICU case volume and decreased in-hospital mortality. In particular, ICUs with a higher percentage (> 75%) of emergency patients showed more pronounced effects, with the odds ratios for in-hospital mortality in the higher case volume quartiles (Q2, Q3, and Q4) being 0.92 (95% credible interval [CI]: 0.88–0.96), 0.70 (95% CI: 0.67–0.73), and 0.78 (95% CI: 0.73–0.83), respectively, compared with the lowest quartile (Q1). Similar trends were observed for various secondary outcomes.

**Conclusions:**

Higher ICU case volumes were significantly associated with lower in-hospital mortality rates in Japanese ICUs predominantly treating critically ill emergency patients. These findings emphasize the importance of ICU specialization and highlight the potential benefits of centralized care for critically ill emergency patients. These findings are potential insights for improving health care policy in Japan and may be valuable in emergency care settings in other countries with similar healthcare systems, after careful consideration of contextual differences.

**Supplementary Information:**

The online version contains supplementary material available at 10.1186/s40560-024-00733-3.

## Background

Previous studies have explored the association between the number of cases and patient outcomes for critical illnesses such as sepsis and trauma and various surgeries, with the expectation that a higher number of cases would have a more favorable effect on patient outcomes [1–8]. Therefore, a positive relationship between case volume and outcome in a broader emergency patient population is expected. However, no such studies have been conducted.

In Japan’s emergency medical care system, critically ill emergency patients are admitted to intensive care units (ICUs) dedicated to emergency patients or to ICUs that also admit critically ill patients whose condition deteriorated while being treated on the general ward and patients after major surgery. These two types of ICUs in Japan exist in roughly equal numbers [9]. In addition to the different nature of each type, the number and proportion of emergency patients admitted to ICUs is expected to vary widely, depending on the individual hospital and the nature of the local healthcare system. Despite the potentially important role these differences could have on patient outcome, no comprehensive study has examined the effect of ICU specialization and case volume on patient outcomes within the Japanese emergency medical care framework. Therefore, the aim of this study was to examine the association between critically ill emergency patient case volumes, specialization, and outcomes by using a nationwide database to provide valuable insights into the optimization of emergency care.

## Methods

### Study design and data

This retrospective cohort study used data from the Japanese Intensive Care PAtient Database (JIPAD), a national registry established by the Japanese Society of Intensive Care Medicine (JSICM) to create a high-quality ICU database. The details of this registry have been previously described [10]. The JIPAD was initiated in 2014, and data have been available since fiscal year (FY) 2015.

Patients aged ≥ 16 years who were registered in the JIPAD between April 1, 2015 and March 31, 2021 included emergency patients admitted directly from the emergency department (ED), emergency patients admitted after surgery, emergency patients transferred from other hospitals, and patients transferred from non-ICU wards or care units within 2 days of emergency admission.

Patients were excluded who were transferred from non-ICU care units or wards after 2 days of emergency admission, had planned admissions, and were admitted to the ICU only for procedures. Facilities with missing information on the ICU staff (e.g., dedicated intensivists and dedicated ICU nurses) and on patients admitted to these facilities, and patients with missing Japan Risk of Death (JROD) scores [11] were excluded because they lacked essential information. Facilities having < 10 eligible patients per year and patients admitted to these facilities were excluded to address heterogeneity in patient care. The JROD score is a prognostic score calibrated for Japanese ICU patients, based on the Acute Physiology and Chronic Health Evaluation III-j scoring system [11, 12].

### Ethics statement

This study was approved by the Institutional Ethics Committee of Kurashiki Central Hospital (approval number: 4266; approval date: November 5, 2023). The committee confirmed that this study adheres to national ethical guidelines and the Declaration of Helsinki. All patients were de-identified, and the need for informed consent was waived.

### Variables

Patient-level variables collected at admission included the JROD score, Sequential Organ Failure Assessment score, age, sex, underlying disease, body mass index (BMI), emergency surgery, cardiac resuscitation before admission, route of admission (i.e., ED, operating room, transfer from another hospital, non-ICU care unit, or ward), and disease group diagnosed at admission. We collected data on various invasive procedures performed in the ICU such as extracorporeal membrane oxygenation (venovenous or venoarterial), invasive mechanical ventilation, and the administration of continuous renal replacement therapy. Additionally, the fiscal years of admission and length of hospitalization were recorded. Facility-level data such as the type of hospital (university hospital or nonuniversity hospital), the proportion of emergency admissions, the number of intensivists and nurses, and the quantity of ICU and hospital beds were also collected.

### Study outcomes

The primary outcome assessed was in-hospital mortality. Secondary outcomes included ICU mortality, 28-day mortality, ventilator-free days (VFDs) 28 days after admission, total length of ICU stay, and length of hospital stay. We defined VFDs as the number of days alive and free of invasive mechanical ventilation during the first 28 days after admission (i.e., 0 days if the patient died within 28 days or received invasive mechanical ventilation for > 28 days) [13].

### Statistical analysis

We divided each ICU by the quartile of the average number of eligible patients admitted per year and described the patient and facility characteristics for each quartile. Categorical data are presented as the number and percentage, and continuous variables are presented as the median and interquartile range (IQR). We calculated the risk-standardized mortality ratio (RSMR) [14] for each ICU by using the number of deaths in each ICU and the JROD score for each patient. We compared each ICU by using a funnel plot of the RSMR.

To account for our two-level hierarchy data structure, we used Bayesian hierarchical generalized linear mixed models with ICU-specific random effects, while adjusting for patient- and ICU-level variables as the fixed effects, and allowing for heterogeneity between ICUs. A random intercept was calculated for each ICU. We estimated an ‘‘empty’’ model (Model 1), which only included each ICU as a random intercept and allowed the detection of in-hospital mortality in various ICUs. The ICU-level random effect of the intercept was assumed to be normally distributed, with a mean value of zero. Thereafter, we estimated the full model (Model 2) to assess the association between case volume and in-hospital mortality by using patient- and ICU-level variables. Logistic regression was applied to in-hospital mortality, ICU mortality, and 28-day mortality. Linear regression models were applied to VFDs at 28 days, total length of ICU stay, and length of hospital stay. Patient-level variables were adjusted for age, sex, JROD score, BMI, cardiac resuscitation before admission, emergency surgery, admission diagnosis, and hospitalization period (FY 2015–2019 or FY 2020–2021). We classified the patients’ BMI into categories appropriate for Asian populations [15]. We adjusted for the type of hospital (university hospital or nonuniversity hospital), number of beds, number of intensivists per ICU bed, number of nurses per ICU bed, and percentage of emergency patients among all admitted patients. The number of beds in each hospital was classified into quartiles. The proportion of emergency patients to all admitted patients was divided into four quadrants, separated by 25%. Each quartile group was stratified, based on the percentage of emergency patients among all patients admitted to each ICU (Model 3), to assess the effects of case volume and specialization on critical emergency patients. We defined the 75% threshold as the “emergency patient-dominant group.” The threshold of 50% or 90% was used for the sensitivity analysis. Markov chain Monte Carlo (MCMC) methods were used to calculate the odds ratios (ORs) or regression coefficients and their corresponding 95% credible intervals (CIs). In the MCMC process, the first 2500 simulations were discarded as the burn-in and the remaining 10,000 simulations were obtained. Normal priors were used for the fixed effects, and noninformative uniform priors were used for the variance of each ICU in the mixed-effects model. The median ORs (MORs) were computed for ICU-level variance [16, 17]. All analyses were performed using the Stata version 16.1 software (Stata, College Station, TX, USA).

## Results

### Patients and ICU characteristics

**Fig. 1:**
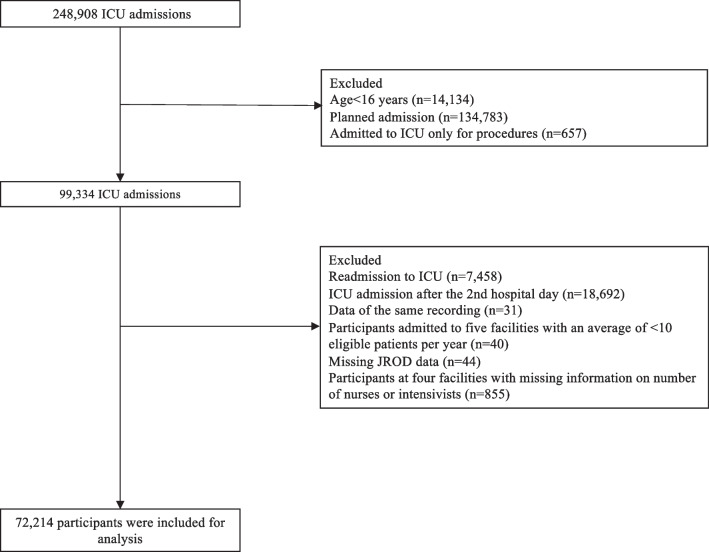
Study flow diagram. The included patients are 16 years or older. They were enrolled in the JIPAD between April 2015 and March 2021 and were admitted immediately to the ICU or the next day after hospital admission. The exclusion criteria applied to facilities missing ICU staff data, patients lacking JROD scores, and facilities with fewer than 10 qualifying patients annually among their patients. *JIPAD* Japanese Intensive Care PAtient Database, *ICU* intensive care unit, *JROD* Japan Risk of Death

We identified 248,908 ICU admission records from 89 ICUs. After applying the exclusion criteria, a total of 80 centers and 72,214 participants were included in the analysis (Fig. 1). Table 1 and Supplementary Table 1 show the patients’ characteristics for each quartile of the number of eligible patients in each ICU. The characteristics of the ICUs for each quartile of the number of patients are described in Table 2. The annual number of eligible admissions was 352 (215.8–469.5) with 152 (118.6–192.3) in the first quartile (Q1), 294 (266.7–318.3) in the second quartile (Q2), 396.6 (391–459.8) in the third quartile (Q3), and 682.5 (541.8–699.3) for the fourth quartile (Q4). A total of 10,704 (14.8%) patients died during hospitalization with a VFD of 23 days.

**Table 1:** Characteristics of participants by quartile

	Total	First quartile	Second quartile	Third quartile	Fourth quartile
	(n = 72,214)	(n = 18,335) (25.4%)	(n = 18,887) (26.2%)	(n = 17,843) (24.7%)	(n = 17,149) (23.8%)
Age (y), median (IQR)	71 (57–80)	71 (58–80)	71 (57–79)	70 (55–79)	72 (60–81)
Sex (male), n (%)	44,534 (61.7)	11,126 (60.68)	11,663 (61.8)	11,173 (62.6)	10,572 (61.7)
BMI category, n (%)					
< 18.5	10,360 (14.7)	2704 (15.1)	2525 (13.6)	2577 (14.8)	2554 (15.3)
≥ 18.5 to < 23	28,081 (39.8)	7136 (39.9)	7501 (40.5)	6760 (38.9)	6684 (40.0)
≥ 23 to < 27.5	22,151 (31.4)	5583 (31.2)	5847 (31.5)	5396 (31.1)	5325 (31.8)
≥ 27.5	9931 (14.1)	2453 (13.7)	2665 (14.4)	2648 (15.2)	2165 (12.9)
JROD, median (IQR)	0.05 (0.02–0.18)	0.05 (0.02–0.17)	0.06 (0.02–0.18)	0.05 (0.01–0.19)	0.05 (0.02–0.17)
Chronic organ insufficiency, n (%)					
Heart failure	1202 (1.7)	505 (2.8)	221 (1.2)	192 (1.1)	284 (1.7)
Respiratory failure	1060 (1.5)	418 (2.3)	226 (1.2)	209 (1.2)	207 (1.2)
Renal dialysis	3639 (5.0)	1006 (5.5)	1080 (5.7)	816 (4.6)	737 (4.3)
Type of hospital, n (%)					
University hospital	28,969 (40.1)	8999 (49.1)	8575 (45.4)	7855 (44.0)	3540 (20.6)
Nonuniversity hospital	43,245 (59.9)	9336 (50.9)	10,312 (54.6)	9988 (56.0)	13,609 (79.4)
Admission source, n (%)					
Operating room	17,060 (23.6)	5912 (32.2)	4018 (21.3)	3813 (21.4)	3317 (19.3)
Emergency department	44,682 (61.9)	8133 (44.4)	12,434 (65.8)	11,960 (67.0)	12,155 (70.9)
Ward	5377 (7.5)	2397 (13.1)	1240 (6.6)	1032 (5.8)	708 (4.1)
Transfer from another hospital	3737 (5.2)	1412 (7.7)	660 (3.5)	824 (4.6)	841 (4.9)
Other care unit	1357 (1.9)	481 (2.6)	534 (2.8)	214 (1.2)	128 (0.8)
Emergency surgery, n (%)	22,421 (31.1)	7506 (40.9)	5614 (29.7)	5110 (28.6)	4191 (24.4)
Diagnosis at ICU admission, n (%)					
Cardiovascular disease	26,880 (37.2)	7384 (40.3)	7087 (37.5)	6563 (36.8)	5846 (34.1)
Respiratory disease	9131 (12.6)	2409 (13.1)	2039 (10.8)	2241 (12.6)	2442 (14.2)
GI and liver disease	9882 (13.7)	3252 (17.7)	2395 (12.7)	1908 (10.7)	2327 (13.6)
Neurologic disease	10,603 (14.7)	2098 (11.4)	3464 (18.3)	2766 (15.5)	2275 (13.3)
Sepsis	2607 (3.6)	674 (3.7)	685 (3.6)	500 (2.8)	748 (4.4)
Trauma	5311 (7.4)	612 (3.3)	1367 (7.2)	1761 (9.9)	1571 (9.2)
After cardiac resuscitation, n (%)	4571 (6.3)	1004 (5.5)	1350 (7.2)	1369 (7.7)	848 (4.9)
IMV during the 1st ICU stay, n (%)	33,333 (46.2)	9039 (49.3)	8436 (44.7)	8365 (46.9)	7493 (43.7)
Venoarterial ECMO, n (%)	1563 (2.2)	498 (2.7)	452 (2.4)	413 (2.3)	200 (1.2)
Venovenous ECMO, n (%)	520 (0.7)	178 (1.0)	137 (0.7)	125 (0.7)	80 (0.5)
CRRT, n (%)	6350 (8.8)	2015 (11.0)	1841 (9.8)	1366 (7.7)	1128 (6.6)
In-hospital mortality, n (%)	10,704 (14.8)	2725 (14.9)	2768 (14.7)	2738 (15.3)	2473 (14.4)

*IQR* interquartile range, *BMI* body mass index, *JROD* Japan Risk of Death, *ICU* intensive care unit, *GI* gastrointestinal, *IMV* invasive mechanical ventilation, *ECMO* extracorporeal membrane oxygenation, *CRRT* continuous renal replacement therapy

**Table 2:** Characteristics of ICUs, based on the quartile of annual eligible patients

	Total	First quartile	Second quartile	Third quartile	Fourth quartile
	(n = 80)	(n = 39)	(n = 19)	(n = 15)	(n = 7)
Type of hospital, n (%)					
University hospital	36 (45.0)	19 (48.7)	9 (47.4)	6 (40.0)	2 (28.6)
Nonuniversity hospital	44 (55.0)	20 (51.3)	10 (52.6)	9 (60.0)	5 (71.4)
Number of eligible patients per year, median (IQR)	352 (215.8–469.5)	152 (118.6–192.3)	294 (266.7–318.3)	396.6 (391–459.8)	682.5 (541.8–699.3)
Hospital beds, median (IQR)	654 (538–832)	697 (550–1044)	613 (500–934)	639 (465–819)	685 (562–1097)
ICU beds, median (IQR)	10.5 (8–14)	10 (6–14)	12 (10–14)	12 (8–17)	14 (10–18)
ICU beds to total number of beds, %, median (IQR)^a^	1.58 (1.11–2.12)	1.34 (1.00–1.76)	1.87 (1.25–2.17)	1.99 (1.45–2.44)	1.98 (1.09–4.40)
Number of ICU nurses, median (IQR)	34.5 (27–47)	30 (24–44)	40 (32–48)	35 (28–53)	45 (33–70)
Number of intensivists, median (IQR)	4 (2.5–10)	4 (2–8)	5 (3–11)	4 (3–10)	7 (4–12)

*ICU* intensive care unit, *IQR* interquartile range

^a^Some hospitals have multiple ICUs registered separately, yet the ratios may not be entirely accurate due to the inability to identify each facility

### Risk-standardized mortality ratio

**Fig. 2:**
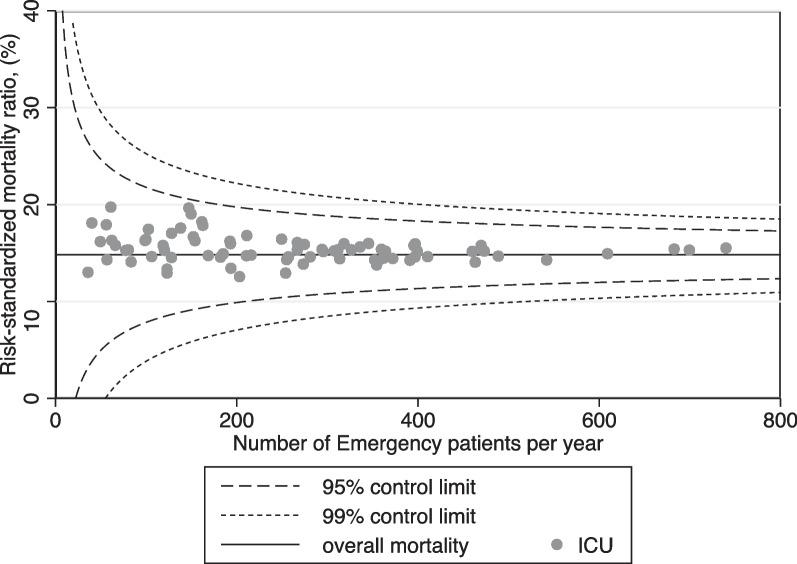
Funnel plots showing risk-standardized mortality rates among ICUs. The overall distribution is presented using the mean mortality ratio (solid line) and the control limits of 95% (dashed line) and 99.8% (dotted line). Each circle represents a single ICU

The RSMR for each ICU are shown in Fig. 2. The variation in the RSMR was higher in ICUs with fewer emergency admissions, especially those with less than 200 admissions.

### In-hospital mortality

The ORs for the in-hospital mortality rates are shown in Table 3 and Supplementary Table 2. In Model 2, higher ICU volumes were associated with decreased in-hospital mortality. We evaluated the association between case volume and in-hospital mortality, adjusted for patient-level and ICU-level variables, and found that the ORs for Q3 and Q4 were 0.92 (95% CI: 0.88–0.95) and 0.93 (95% CI: 0.88–0.99), respectively, indicating decreased in-hospital mortality, compared with Q1. In Model 1, the MOR is 1.40 (95% CI: 1.32–1.49), indicating a significant variation in in-hospital mortality at the ICU level. In Model 2, adjusted for patient-level and ICU-level variables, we found a smaller MOR of 1.07 (95% CI: 1.02–1.12).

**Table 3:** Odds ratios for hospital mortality

	Model 1	Model 2	Model 3
	OR (95% CI)	OR (95% CI)	OR (95% CI)
Random effects			
Median odds ratio	1.40 (1.32–1.49)	1.07 (1.02–1.12)	1.07 (1.04–1.12)
ICU level variance, (SD)	0.12 (0.02)	0.0056 (0.0038)	0.0054 (0.0031)
Fixed effects			
ICU-level variables			
Quartile of ICU admissions			
First quartile		Ref.	
Second quartile		0.96 (0.92–1.01)	
Third quartile		0.92 (0.88–0.95)	
Fourth quartile		0.93 (0.88–0.99)	
Emergency patients as % of total ICU admissions			
< 25		Ref.	
≥ 25 to < 50		0.97 (0.93–1.02)	
≥ 50 to < 75		0.99 (0.95–1.02)	
≥ 75		0.83 (0.80–0.86)	
Admission quartiles and percentage of emergency patients			
First quartile, < 75%			Ref.
Second quartile, < 75%			0.95 (0.92–0.98)
Third quartile, < 75%			0.93 (0.89–0.98)
Fourth quartile, < 75%			0.92 (0.89–0.96)
First quartile, ≥ 75%			NA^a^
Second quartile, ≥ 75%			0.92 (0.88–0.96)
Third quartile, ≥ 75%			0.70 (0.67–0.73)
Fourth quartile, ≥ 75%			0.78 (0.73–0.83)
Type of hospital			
University hospital		Ref.	Ref.
Non-university hospital		1.00 (0.97–1.04)	0.98 (0.94–1.01)
Number of intensivists per ICU bed		1.06 (1.03–1.10)	1.05 (1.01–1.10)
Number of nurses per ICU bed		0.98 (0.96–1.00)	0.98 (0.96–1.00)
Quartile of hospital beds			
First quartile		Ref.	Ref.
Second quartile		1.02 (1.00–1.04)	1.01 (0.96–1.07)
Third quartile		1.02 (0.98–1.07)	1.05 (1.00–1.10)
Fourth quartile		0.99 (0.96–1.01)	0.99 (0.95–1.03)

Odds ratios were calculated using a multilevel logistic regression model, allowing for a random effect (a random intercept) model for each ICU. We adjusted ICU-level and patient-level variables as follows: age, sex, BMI (< 18.5, 18.5 to 23, 23 to 27.5, ≥ 27.5), the Japan Risk of Death score, diagnoses at admission and after cardiac resuscitation, emergency surgery, hospitalization period (from FY 2015 through FY 2019, from FY 2020 through FY 2021), number of nurses per ICU beds, number of intensivists per ICU beds, quartile of hospital beds, and type of hospital (university hospital or non-university hospital). The odds ratios for in-hospital mortality associated with patient-level variables are detailed in the Supplementary Table 2

*SD* standard deviation, *OR* odds ratio, *CI* credible interval, *Ref.* reference, *NA* not applicable

^a^All first quartile participants were enrolled in facilities with less than 75% emergency patients

### Secondary outcomes

Results for the secondary outcomes are shown in Supplementary Table 3. Q4 had ORs of 1.32 (95% CI: 1.24–1.41) and 1.12 (95% CI: 1.09–1.15) for ICU deaths and 28-day deaths, respectively. These values remained large after adjusting for patient-level and ICU-level variables in Model 2, but were inconsistent with the results for in-hospital mortality. We found that the case volume did not affect VFDs, ICU length of stay, or the reduced hospital length of stay in Q3 and Q4.

### Stratified analyses

**Fig. 3:**
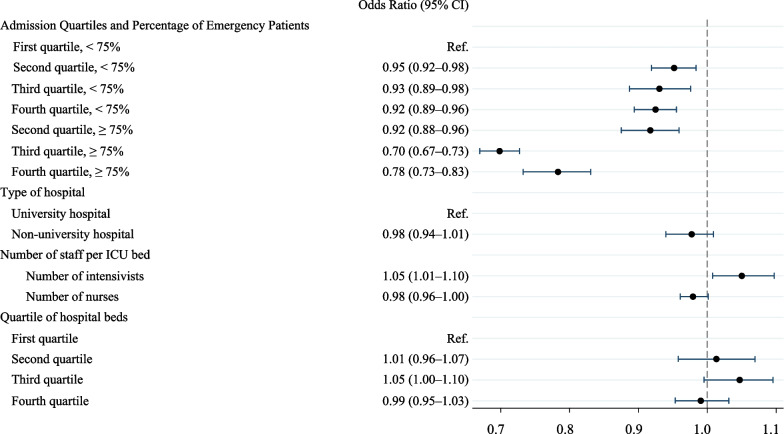
In-hospital mortality, stratified by the number of ICU admissions and percentage of emergency patients. Odds ratios were calculated using a multilevel logistic regression model, thereby allowing for a random effect (i.e., random intercept) model for each ICU. We adjusted ICU-level and patient-level variables, as follows: age, sex, BMI (< 18.5, 18.5–23, 23–27.5, and ≥ 27.5), JROD score, diagnosis at admission and after cardiac resuscitation, emergency surgery, hospitalization period (from FY 2015 through FY 2019 and from FY 2020 through FY 2021), number of nurses per ICU beds, number of intensivists per ICU beds, quartile of hospital beds, and type of hospital (university hospital or nonuniversity hospital). *ICU* intensive care unit, *JROD* Japan Risk of Death, *BMI* body mass index, *FY* fiscal year, *Ref.* reference

In Model 3, the quartiles were further stratified and examined, based on the percentage of emergency patients (i.e., > 75%). In Q1, no ICUs were included in the “emergency patient-dominant group” stratum. The findings of the study suggests that case volume had a larger effect on ICUs with an “emergency patient-dominant group” strata, as indicated by the lack of overlap in their respective 95% CI ranges (Fig. 3). The ORs for Q2, Q3, and Q4 in this stratum were 0.92 (95% CI: 0.88–0.96), 0.70 (95% CI: 0.67–0.73), and 0.78 (95% CI: 0.73–0.83), respectively. In-hospital mortality rates were lower in Q2, Q3, and Q4 than in Q1, even in ICUs with emergency patient ratios of < 75%. Sensitivity analyses were similar when the thresholds were set at 90% and 50% (Supplementary Table 4).

The stratified analysis of secondary outcomes is shown in Supplementary Table 5. For the “emergency patient-dominant group,” Q4 showed a reduction in ICU mortality with an OR of 0.77 (95% CI; 0.73–0.82), indicating heterogeneity in the association between case volume and outcome, depending on the frequency of emergency patients.

## Discussion

This study assessed the effects of case volume and specialization on the outcomes of critically ill emergency patients by using a comprehensive ICU patient database. The results revealed that higher ICU case volumes were associated with lower in-hospital mortality rates, particularly in ICUs with higher proportions of emergency patients.

This association is consistent with the findings of previous studies [2, 3, 7, 8, 18] conducted on other certain emergencies, supporting the learning curve hypothesis [18]. Another possible mechanism is that the ICUs in the lowest quartile (Q1) had fewer ICU beds relative to total hospital beds (Table 2), suggesting limited resources. Although these ICUs may treat more severely ill patients, the impact of bed count is minimal because adjustments were made for illness severity and staff number. Our analysis also revealed a nonlinear association between case volume and patient outcomes. This U-shaped association was more evident for ICU mortality and 28-day mortality, suggesting that a similar mechanism may exist as that described in a previous studies [19, 20] in which an excess case volume was negatively associated with mortality. However, as shown in Supplementary Table 5, we observed differences in short-term mortality rates and hospital mortality rates in Q4, depending on the proportion of emergency patients. This indicates that the effect of case volume on short-term mortality is heterogeneous across the proportion of emergency patients in the ICU.

Furthermore, the stratified analysis by proportion of emergency patients showed a more obvious reduction in in-hospital mortality in ICUs with a predominantly emergency patient population, which may be because of the positive impact of ICU specialization. These ICUs may be well resourced and experienced in the treatment of emergency conditions, which may lead to better patient outcomes.

In this study, the MOR for in-hospital mortality was low (MOR 1.07; 95% CI: 1.02–1.12), indicating little variation in in-hospital mortality among ICUs. However, the MOR for short-term mortality, especially ICU mortality, was significantly higher (MOR 1.36; 95% CI: 1.27–1.46), suggesting a notable disparity in short-term outcomes, which were potentially influenced by ICU-level and patient-level variables. The MOR is defined as the median value of the OR between the highest and lowest risk clusters; if two clusters are chosen at random, the MOR indicates the increased risk (in median) of moving to another higher-risk cluster [16].

The MOR for ICU mortality increased substantially, suggesting a significant variation in short-term mortality risk across ICUs, which cannot be fully explained by ICU- or patient-level variables. These MOR results may have been derived from differences between the ICUs that were not captured in this dataset. Factors that may have created variations include ICU practices and protocols (e.g., differences in treatment protocols, staffing, and available resources), admission criteria (e.g., variation in patient admission criteria that may affect the risk profile of ICU patients), discharge criteria (affecting the length of ICU stay), facility characteristics (e.g., lack of high-dependency care units, which may affect admission and discharge criteria), and regional differences in the provision and use of critical care beds [21]. These findings indicate that further investigation of the factors affecting patient outcomes in the ICUs is required.

The RSMR for in-hospital mortality for each ICU (Fig. 2) could be appropriately compared with that of the entire population by using a funnel plot [14], showing the variation in the RSMR for ICUs with fewer emergency admissions. This finding suggests disparities in resources, quality of care, or patient population characteristics. This disparity was supported by the multilevel analysis (Model 2), which showed increased in-hospital mortality in ICUs with fewer than 200 emergency admissions per year (Q1), after adjusting for patient characteristics and ICU resources. Higher-case-volume ICUs may have lower RSMRs, possibly because of factors such as experienced staff, effective protocols, and resource availability.

The RSMR is a crucial indicator of quality of care but must be interpreted in conjunction with other indicators, such as the length of stay and readmission rates, for a comprehensive view of ICU performance. When calculating the RSMR, the method of risk adjustment must be considered to avoid misleading results—particularly if certain high-risk patient populations are inadequately accounted for. We improved the reliability of our results by using the JROD score [11], a newly developed index for intensive care patients in Japan. However, missing values or reporting bias when calculating the RSMR could affect the accuracy and reliability of the results.

One strength of this study was the use of the JIPAD, which registers various ICUs nationwide and regularly undertakes efforts to maintain data accuracy [22]. It is the most reliable database for ICUs in Japan in terms of size, reliability, and precision. Therefore, we believe that the participants and facilities in this study represent a highly representative population of emergency patients requiring intensive care in Japan.

This study has some limitations. Each facility in the JIPAD is anonymized; therefore, we classified the participating facilities, based on the ratio of emergencies to admitted patients. Second, a possibility of selection bias existed because five of nine centers were excluded because they had a small number of potentially eligible patients, they treated primarily pediatric patients, and were highly heterogeneous, whereas the other four centers lacked information on the number of intensivists and nurses. Although information on the number of intensivists and nurses was lacking, the small number of excluded patients had little impact on the results. Third, participation in the JIPAD was voluntary; therefore, the participating ICUs may have been more proactive in improving the quality of care. ICUs with larger case volumes or a higher proportion of emergency patients are more likely to participate in the JIPAD, which may cause further selection bias. Nevertheless, analyzing a homogeneous population increases the validity of comparisons and the reliability of statistical analysis. Furthermore, caution should be exercised when generalizing the results because these ICUs may not be fully representative of all ICUs in Japan. Fourth, we were unable to assess the proficiency or years of experience of the ICU staff. In Japan, intensivists typically have a background in emergency medicine or anesthesia [23]. We also could not assess differences in the background of intensivists. These differences could have influenced the patient outcomes, and therefore require further investigation into the effect of the expertise and training of ICU staff on patient outcomes. A fifth limitation is differences in healthcare systems. Extrapolating the results of this study to other countries may be limited by differences in healthcare systems, especially in ICU settings. However, extrapolation to other countries may be possible. Even after considering the effects of these differences, the results of this study may be relevant beyond the Japanese healthcare system. For instance, a comparable mechanism may be responsible for favorable patient outcomes in the emergency department intensive care unit (ED-ICU) system in the United States [24] or in ICUs where emergency physicians led operations in South Korea [25]. Specifically, this improvement in outcomes can be attributed to the shortened time to ICU admission for emergency patients, effective coordination between the ED and ICU, reduced length of stay in the ED, and a comprehensive understanding of the patients’ condition. Nevertheless, direct comparisons among different healthcare systems should be made with caution. Finally, the utilization of critical care and emergency medical systems in Japan was affected by the COVID-19 pandemic since April 2020 (FY 2020 and beyond) [26–28], which may have an impact on patient outcomes. Thus, we categorized data entry into two periods: FY 2015–2019 and FY 2020–2021. Future research could potentially focus on exploring the impact of different ICU characteristics and healthcare reimbursement classifications on critically ill patient outcomes. This research could involve examining factors, such as ICU size, patients’ demographics, and financial incentives within the reimbursement system, to better understand how these factors may influence care quality.

## Conclusions

Higher case volumes and specialization of critically ill emergency patients are associated with a lower risk of in-hospital mortality. Based on these results, we recommend that critically ill emergency patients be centralized and admitted to specialized ICUs for emergency patients to optimize the emergency care system. Meanwhile, significant variability existed among ICUs in short-term mortality. Future studies focusing on regional differences and staff specialization are needed to determine the causes contributing to this variation.

### Supplementary Information

Supplementary Material 1.

## Data Availability

The author’s agreement with the JIPAD does not allow publishing the data used for this manuscript or sharing it with others. The JIPAD Working Group would cooperate in case any fraud or forgery is suspected in manuscripts in which JIPAD data are used.

## Abbreviations

BMI: Body mass index
CI: Credible interval
ED: Emergency department
FY: Fiscal year
ICU: Intensive care unit
IQR: Interquartile range
JROD: Japan Risk of Death
JIPAD: Japanese Intensive Care PAtient Database
JSICM: Japanese Society of Intensive Care Medicine
MOR: Median odds ratio
OR: Odds ratio
RSMR: Risk-standardized mortality ratio
VFD: Ventilator-free day
