# Supplementary material for: Case volume and specialization in critically ill emergency patients: a nationwide cohort study in Japanese ICUs
Source: J Intensive Care. 2024 May 17;12:20. doi: 10.1186/s40560-024-00733-3 (PMC11100151; doi:10.1186/s40560-024-00733-3)
Supplement: Supplementary file 1 — Supplementary Material 1. [file 40560_2024_733_MOESM1_ESM.docx]

| Supplementary Table 1. Characteristics of the participants, based on quartile | | | | | | | | | | |  |
| --- | --- | --- | --- | --- | --- | --- | --- | --- | --- | --- | --- |
|  |  | Total |  | First quartile |  | Second quartile |  | Third quartile |  | Fourth quartile |  |
|  |  | (n=72,214) |  | (n=18,335) (25.4%) |  | (n=18,887) (26.2%) |  | (n=17,843) (24.7%) |  | (n=17,149) (23.8%) |  |
| BMI, median (IQR) |  | 22.5 (19.9–25.4) |  | 22.5 (19.8–25.4) |  | 22.6 (20.0–25.5) |  | 22.6 (19.9–25.6) |  | 22.5 (19.8–25.3) |  |
| SOFA, median (IQR) |  | 5 (3–8) |  | 6 (3–9) |  | 6 (3–9) |  | 5 (3–8) |  | 5 (3–8) |  |
| Chronic organ insufficiency, n (%) |  |  |  |  |  |  |  |  |  |  |  |
| Heart failure |  | 1202 (1.7) |  | 505 (2.8) |  | 221 (1.2) |  | 192 (1.1) |  | 284 (1.7) |  |
| Respiratory failure |  | 1060 (1.5) |  | 418 (2.3) |  | 226 (1.2) |  | 209 (1.2) |  | 207 (1.2) |  |
| Liver failure |  | 454 (0.6) |  | 142 (0.8) |  | 115 (0.6) |  | 92 (0.5) |  | 105 (0.6) |  |
| Liver cirrhosis |  | 1243 (1.7) |  | 289 (1.6) |  | 293 (1.6) |  | 307 (1.7) |  | 354 (2.1) |  |
| Solid tumor with metastasis |  | 1687 (2.3) |  | 599 (3.3) |  | 382 (2.0) |  | 349 (2.0) |  | 357 (2.1) |  |
| Immunosuppression |  | 2715 (3.8) |  | 1129 (6.2) |  | 658 (3.5) |  | 483 (2.7) |  | 445 (2.6) |  |
| Renal dialysis |  | 3639 (5.0) |  | 1006 (5.5) |  | 1080 (5.7) |  | 816 (4.6) |  | 737 (4.3) |  |
| Hematologic malignancy |  | 718 (1.0) |  | 238 (1.3) |  | 138 (0.7) |  | 169 (1.0) |  | 173 (1.0) |  |
| Diagnosis at ICU admission, n (%) |  |  |  |  |  |  |  |  |  |  |  |
| Cardiovascular disease |  | 26,880 (37.2) |  | 7384 (40.3) |  | 7087 (37.5) |  | 6563 (36.8) |  | 5846 (34.1) |  |
| Respiratory disease |  | 9131 (12.6) |  | 2409 (13.1) |  | 2039 (10.8) |  | 2241 (12.6) |  | 2442 (14.2) |  |
| GI and liver disease |  | 9882 (13.7) |  | 3252 (17.7) |  | 2395 (12.7) |  | 1908 (10.7) |  | 2327 (13.6) |  |
| Neurologic disease |  | 10,603 (14.7) |  | 2098 (11.4) |  | 3464 (18.3) |  | 2766 (15.5) |  | 2275 (13.3) |  |
| Sepsis |  | 2607 (3.6) |  | 674 (3.7) |  | 685 (3.6) |  | 500 (2.8) |  | 748 (4.4) |  |
| Trauma |  | 5311 (7.4) |  | 612 (3.3) |  | 1367 (7.2) |  | 1761 (9.9) |  | 1571 (9.2) |  |
| Endocrinology disease |  | 3890 (5.4) |  | 665 (3.6) |  | 903 (4.9) |  | 1252 (7.0) |  | 1070 (6.2) |  |
| Hematology disease |  | 321 (0.4) |  | 93 (0.5) |  | 71 (0.4) |  | 76 (0.4) |  | 81 (0.5) |  |
| Other |  | 3589 (5.0) |  | 1148 (6.3) |  | 876 (4.6) |  | 776 (4.4) |  | 789 (4.6) |  |
| ICU mortality, n (%) |  | 5782 (8.0) |  | 1467 (8.0) |  | 1414 (7.5) |  | 1453 (8.1) |  | 1448 (8.4) |  |
| 28-day mortality, n (%) |  | 8615 (11.9) |  | 2048 (11.2) |  | 2262 (12.0) |  | 2217 (12.4) |  | 2088 (12.2) |  |
| VFD at 28days, days, median (IQR) |  | 23 (12–26) |  | 23 (12–26) |  | 23 (11–26) |  | 23 (11–26) |  | 23 (12–26) |  |
| Hospital LOS, days, median (IQR) |  | 19 (11–34) |  | 21 (12–37) |  | 20 (11–35) |  | 18 (10–34) |  | 18 (11–31) |  |
| ICU LOS, total, days, median (IQR) |  | 4 (2–7) |  | 4 (2–8) |  | 4 (2–7) |  | 4 (2–7) |  | 4 (2–7) |  |
| ICU LOS, first, days, median (IQR) |  | 4 (2–7) |  | 4 (2–8) |  | 4 (2–7) |  | 3 (2–6) |  | 4 (2–7) |  |
|  |  |  |  |  |  |  |  |  |  |  |  |
| BMI, body mass index; IQR, interquartile range; SOFA, Sequential Organ Failure Assessment; ICU, intensive care unit; GI, gastrointestinal; VFD, ventilator-free days; LOS, length of stay | | | | | | | | | | |  |
|  |  |  |  |  |  |  |  |  |  |  |  |
|  |  |  |  |  |  |  |  |  |  |  |  |

| Supplementary Table 2. Odds ratios for hospital mortality | | | | |
| --- | --- | --- | --- | --- |
|  |  | Model 2 |  | Model 3 |
|  |  | OR (95% CI) |  | OR (95% CI) |
| Patient-level variable |  |  |  |  |
| Age |  | 1.01 (1.00–1.01) |  | 1.01 (1.01–1.01) |
| Sex, male |  | 1.08 (1.06–1.11) |  | 1.08 (1.03–1.11) |
| JROD |  | 441.37 (403.59–484.05) |  | 431.95 (421.78–442.07) |
| BMI |  |  |  |  |
| ≥18.5 to <23 |  | Ref. |  | Ref. |
| <18.5 |  | 1.33 (1.27–1.39) |  | 1.35 (1.29–1.42) |
| ≥23 to <27.5 |  | 1.13 (1.10–1.17) |  | 1.12 (1.08–1.15) |
| ≥27.5 |  | 1.19 (1.14–1.23) |  | 1.18 (1.14–1.21) |
| Diagnosis at ICU admission |  |  |  |  |
| Cardiovascular disease |  | Ref. |  | Ref. |
| Respiratory disease |  | 1.88 (1.84–1.92) |  | 1.90 (1.83–1.97) |
| Gastroenterology disease |  | 1.24 (1.20–1.29) |  | 1.26 (1.22–1.31) |
| Neurologic disease |  | 1.12 (1.07–1.16) |  | 1.13 (1.08–1.19) |
| Sepsis |  | 1.20 (1.13–1.26) |  | 1.20 (1.14–1.27) |
| Trauma |  | 1.67 (1.63–1.72) |  | 1.68 (1.62–1.77) |
| Endocrinology disease |  | 0.78 (0.73–0.82) |  | 0.78 (0.74–0.82) |
| Hematology disease |  | 2.39 (2.31–2.48) |  | 2.36 (2.22–2.48) |
| Other |  | 1.32 (1.26–1.37) |  | 1.31 (1.27–1.36) |
| After cardiac resuscitation |  | 1.69 (1.65–1.73) |  | 1.73 (1.58–1.88) |
| Emergency surgery |  | 0.99 (0.93–1.04) |  | 0.99 (0.97–1.02) |
| Hospitalization Period |  |  |  |  |
| From FY 2015 through FY 2019 |  | Ref. |  | Ref. |
| From FY 2020 through FY 2021 |  | 1.01 (0.97–1.04) |  | 1.02 (0.99–1.05) |
|  |  |  |  |  |
| Odds ratios were calculated using a multilevel logistic regression model, allowing for a random effect (a random intercept) model for each ICU. We adjusted the ICU-level and patient-level variables for the following: age, sex, BMI (<18.5, 18.5–23, 23–27.5, and ≥27.5), the Japan Risk of Death score, diagnosis at admission and after cardiac resuscitation, emergency surgery, hospitalization period (from FY 2015 through FY 2019 and from FY 2020 through FY 2021), number of nurses per ICU beds, number of intensivists per ICU beds quartile of hospital beds, and type of hospital (university hospital or nonuniversity hospital).  JROD, Japan Risk of Death; BMI, body mass index; FY, fiscal year; SD, standard deviation; OR, odds ratio; CI, credible interval; Ref., reference | | | | |

| Supplementary Table 3. Secondary outcomes, based on the quartile of ICU admissions | | | | | | | | | | |
| --- | --- | --- | --- | --- | --- | --- | --- | --- | --- | --- |
|  |  | ICU mortality |  | 28-Day mortality |  | 28-Day ventilator-free days |  | ICU LOS, days |  | Hospital LOS, days |
|  |  | OR (95% CI) |  | OR (95% CI) |  | Regression coefficient  (95% CI) |  | Regression coefficient  (95% CI) |  | Regression coefficient  (95% CI) |
| Quartile of ICU Admissions |  |  |  |  |  |  |  |  |  |  |
| First quartile |  | Ref. |  | Ref. |  | Ref. |  | Ref. |  | Ref. |
| Second quartile |  | 0.97 (0.91–1.02) |  | 1.06 (1.03–1.10) |  | 0.25 (-0.53–1.07) |  | -0.79 (-2.48–0.3) |  | -1.27 (-3.48–0.88) |
| Third quartile |  | 0.84 (0.80–0.88) |  | 0.93 (0.89–0.97) |  | 0.54 (-0.31–1.53) |  | -0.82 (-1.98–0.2) |  | -2.86 (-5.15–-0.57) |
| Fourth quartile |  | 1.32 (1.24–1.41) |  | 1.12 (1.09–1.15) |  | -0.02 (-1.13–1.24) |  | -0.75 (-2.42–0.75) |  | -7.71 (-10.47–-4.67) |
|  |  |  |  |  |  |  |  |  |  |  |
| OR, odds ratio; CI, credible interval; LOS, length of stay | | | | | | | | | | |
|  |  |  |  |  |  |  |  |  |  |  |

| Supplementary Table 4. Sensitivity analyses | | | | | | | | |  |
| --- | --- | --- | --- | --- | --- | --- | --- | --- | --- |
|  |  | Quartile of ICU admissions | | | | | | |  |
|  |  | First quartile |  | Second quartile |  | Third quartile |  | Fourth quartile | |
|  |  | OR (95% CI) |  | OR (95% CI) |  | OR (95% CI) |  | OR (95% CI) | |
| Emergency patients as a percentage of total ICU admissions |  |  |  |  |  |  |  |  | |
| <50% |  | Ref. |  | 0.91 (0.88–0.94) |  | 0.93 (0.88–0.98) |  | 0.94 (0.89–0.98) | |
| ≥50% |  | 0.88 (0.85–0.92) |  | 1.00 (0.94–1.07) |  | 0.82 (0.78–0.86) |  | 0.86 (0.82–0.89) | |
|  |  |  |  |  |  |  |  |  | |
| <90% |  | Ref. |  | 0.97 (0.94–1.02) |  | 0.93 (0.89–0.97) |  | 0.93 (0.90–0.97) | |
| ≥90% |  | NA ^a^ |  | 0.86 (0.84–0.89) |  | 0.61 (0.57–0.67) |  | 0.78 (0.75–0.82) | |
| OR, odds ratio; CI, credible interval; NA, not applicable  ^a^ All first quartile participants were enrolled in facilities with less than 90% emergency patients. | | | | | | | | |  |
|  |  |  |  |  |  |  |  |  |  |

| Supplementary Table 5. Secondary outcomes, based on admission quartile and percentage of emergency patients | | | | | | | | | | |
| --- | --- | --- | --- | --- | --- | --- | --- | --- | --- | --- |
|  |  | ICU mortality |  | 28-Day mortality |  | 28-day Ventilator free days |  | ICU LOS, days |  | Hospital LOS, days |
|  |  | OR (95% CI) |  | OR (95% CI) |  | Regression coefficient |  | Regression coefficient |  | Regression coefficient |
|  |  |  |  |  |  | (95% CI) |  | (95% CI) |  | (95% CI) |
| Admission quartile and percentage of emergency patients |  |  |  |  |  |  |  |  |  |  |
| First quartile, <75% |  | Ref. |  | Ref. |  | Ref. |  | Ref. |  | Ref. |
| Second quartile, <75% |  | 0.89 (0.86–0.92) |  | 1.06 (1.01–1.12) |  | 0.44 (-0.37–1.22) |  | -0.97 (-1.97–0.19) |  | -1.09 (-3.18–1.01) |
| Third quartile, <75% |  | 0.73 (0.70–0.76) |  | 0.98 (0.95–1.00) |  | 0.68 (-0.1–1.61) |  | -1.49 (-2.27–-0.53) |  | -2.01 (-4.30–0.20) |
| Fourth quartile, <75% |  | 1.25 (1.21–1.30) |  | 1.11 (1.04–1.18) |  | 0.16 (-0.89–1.49) |  | -0.40 (-1.71–0.84) |  | -6.67 (-10.38–-3.73) |
| First quartile, ≥75% |  | NA^a^ |  | NA ^a^ |  | NA ^a^ |  | NA ^a^ |  | NA ^a^ |
| Second quartile, ≥75% |  | 0.88 (0.82–0.93) |  | 1.08 (1.03–1.13) |  | 0.78 (-0.58–2.13) |  | -0.57 (-2.60–1.11) |  | 0.28 (-3.08–4.07) |
| Third quartile, ≥75% |  | 0.84 (0.81–0.88) |  | 0.72 (0.67–0.78) |  | 1.20 (-0.36–2.79) |  | -1.45 (-3.88–1.25) |  | -3.05 (-7.18–0.64) |
| Fourth quartile, ≥75% |  | 0.77 (0.73–0.82) |  | 0.97 (0.93–1.02) |  | 0.61 (-1.29–2.3) |  | -1.13 (-3.63–2.28) |  | -6.16 (-11.88–-1.47) |
|  |  |  |  |  |  |  |  |  |  |  |
| OR, odds ratio; CI, credible interval; NA, not applicable  ^a^ All first quartile participants were enrolled in facilities with less than 75% emergency patients. | | | | | | | | | | |
|  |  |  |  |  |  |  |  |  |  |  |
